# The diagnostic and prognostic importance of oxidative stress biomarkers and acute phase proteins in Urinary Tract Infection (UTI) in camels

**DOI:** 10.7717/peerj.1363

**Published:** 2015-11-05

**Authors:** Wael M. El-Deeb, Sébastien Buczinski

**Affiliations:** 1 Department of Clinical Studies, College of Veterinary Medicine and Animal Resources, King Faisal University, Al-Ahsa, Saudi Arabia; 2 Department of Veterinary Medicine, Infectious Diseases and Fish Diseases, Faculty of Veterinary Medicine, Mansoura University, Mansoura, Egypt; 3 Département des Sciences Cliniques, Faculté de Médecine Vétérinaire, Université de Montréal, Saint-Hyacinthe, Canada

**Keywords:** Urinary, Camel, Infections, Malondialdehyde, Serum Amyloid A

## Abstract

The present study aimed to investigate the diagnostic and prognostic importance of oxidative stress biomarkers and acute phase proteins in urinary tract infection (UTI) in camels. We describe the clinical, bacteriological and biochemical findings in 89 camels. Blood and urine samples from diseased (*n* = 74) and control camels (*n* = 15) were submitted to laboratory investigations. The urine analysis revealed high number of RBCS and pus cells. The concentrations of serum and erythrocytic malondialdehyde (sMDA & eMDA), Haptoglobin (Hp), serum amyloid A (SAA), Ceruloplasmin (Cp), fibrinogen (Fb), albumin, globulin and interleukin 6 (IL-6) were higher in diseased camels when compared to healthy ones. Catalase, super oxide dismutase and glutathione levels were lower in diseased camels when compared with control group. Forty one of 74 camels with UTI were successfully treated. The levels of malondialdehyde, catalase, super oxide dismutase, glutathione, Hp, SAA, Fb, total protein, globulin and IL-6 were associated with the odds of treatment failure. The MDA showed a great sensitivity (Se) and specificity (Sp) in predicting treatment failure (Se 85%/Sp 100%) as well as the SAA (Se 92%/Sp 87%) and globulin levels (Se 85%/Sp 100%) when using the cutoffs that maximizes the sum of Se + Sp. Multivariate logistic regression analysis revealed that two models had a high accuracy to predict failure with the first model including sex, sMDA and Hp as covariates (area under the receiver operating characteristic curve (AUC) = 0.92) and a second model using sex, SAA and Hp (AUC = 0.89). Conclusively, the oxidative stress biomarkers and acute phase proteins could be used as diagnostic and prognostic biomarkers in camel UTI management. Efforts should be forced to investigate such biomarkers in other species with UTI.

## Introduction

Urinary tract infection (UTI) exists when bacteria adhere, multiply, and persist in a portion of the urinary tract. UTI causes vascular damage to the urinary bladder and decrease the competence of the kidney’s functions, with subsequent conflicts in protein, acid–base, water and solute homeostasis and in the excretion of metabolic end products. When the kidneys can no longer regulate body fluid and solute composition, renal failure occurs and consequently loss of affected animals (Carlton & Mc Gavin, 2001; McGavin & Zachary, 2007; Radostits et al., 2007).

The vulva may play an important role as the portal of entry of bovine urinary infection. Conditions that lead to damage to mucosa in the lower portion of the urinary tract, such as post-parturient diseases or catheterization, may predispose the cow to pyelonephritis (Markusfeld et al., 1989; Rebhun et al., 1989). Cystitis, urethritis and pyelonephritis in cattle most commonly result from ascending urinary tract infection with *Corynebacterium renale*, *Corynebacterium cystidis*, *Corynebacterium pilosum* or *Escherichia coli* (Rebhun et al., 1989; Mills-Wallace et al., 1990; Yeruham et al., 1999; Yeruham et al., 2006). Less common causative organisms include various coliform species (Mills-Wallace et al., 1990).

Cystitis and urethritis are more common in the female camel because of a shorter urethra and the possibility of retrograde invasion by bacteria (Fowler, 1999). The most important predisposing factors for cystitis are ureterolithiasis, bladder paresis and urine stagnation. The bacterial infection cause cystitis mainly comes from ascending or descending route or may also occur by expansion from neighboring organs (Sastry, 1999).

Bacterial infection of the lower urinary tract is usually associated with signs of pollakiuria, dysuria, stranguria, hematuria, and inappropriate urination (Bartges, 2007).

Urine analysis is one of the most important diagnostic tests that can help localize disease, determine causes of discolored urine and identify inflammatory diseases of the urinary system (Pugh, 2002). Urine culture is very essential to determine the type of bacterial infection (Radostits et al., 2007); however, the culture takes at least 2 days to get a result with subsequent delay in the onset of treatment.

A variety of oxidation products are found in urine and thought to mirror local and systemic oxidative stress (Kirschbaum, 2001). Acute terms of various diseases accompany many inflammatory conditions and influence the endogenous antioxidant enzyme activities. UTI may cause an oxidative stress, and also the antioxidant enzymes measured quantitatively were depleted in response to oxidative stress (Kurutas et al., 2005). Kirschbaum (2001) reported that total antioxidant enzyme activity was lower in patients with acute renal disease compared to those of control urine specimens. UTI may cause oxidative stress by consuming urinary antioxidant enzymes and it is possible to say that urinary antioxidant enzymes are not enough to prevent the oxidative stress in UTI (Kurutas et al., 2005). The authors declared that, overproduction of free radicals generated during infection may lead to the low levels of antioxidant enzymes. Urinary malondialdehyde (MDA) is found in increased quantities in some diseases such as thalassemia, renal failure, and pancreatic disease (Kang et al., 2001). Urinary MDA level was 4.75 times higher in positive urine cultures compared to negative urine cultures and may indicate the existence of oxidative stress (Kurutas et al., 2005). The same authors stated that MDA test can be obtained before the results of cultures taken in urinary tract infection; it may be used as an ancillary diagnostic tool and may contribute to the initiation of treatment without waiting for the culture results.

Serum amyloid-A (SAA) and C-reactive protein (CRP) appear to be the most reliable markers for antimicrobial therapy monitoring in patients with urinary tract infections (Casl et al., 1993). Increased levels of SAA expression within the bladder wall versus the urothelium in mice with UTI suggest that infiltrating immune effector cells and resident host cells within this compartment are primary contributors to SAA production during a UTI (Erman et al., 2012). The authors mentioned that enhanced levels of SAA1 expression in response to Uropathogenic Escherichia coli (UPEC) within the urinary tract were also observed systemically, being detected in the liver and transiently within the serum of infected mice. Direct inoculation of UPEC into the peritoneum also increased levels of SAA1 and SAA3 within both the liver and general circulation, with only SAA3 increased in the bladder wall and urothelium. Although the physiological role of SAA during a UTI remains to be tested *in vivo*, the robust localized and systemic amplification of SAA in response to infection with UPEC suggests a critical role for this acute phase protein as a host defense against UTI.

To the best of the authors’ knowledge, there is no data concerning the diagnostic and prognostic role of oxidative stress biomarkers and acute phase response in cases of UTI in camels, which is the main objective of this study.

## Materials and Methods

### Animals

Initially a total of 91 camels (43 male and 48 female) were used in this consecutive cohort study. Camels were clinically investigated in the veterinary teaching hospital, King Faisal University, Saudi Arabia. The project was ethically approved by the Deanship of Scientific Research, King Faisal University (number 130031). According to clinical examination, and the laboratory analysis, the camels were categorized into 2 groups. The first group of camels comprised healthy individuals (*n* = 15; control group) while the second group were camels with clinical features consistent with UTI (*n* = 76; UTI group). The selection of control group was based on clinical and laboratory examination of urine and urine culture. The healthy camels were examined in the hospital for a routine examination before breeding season. The UTI camels were confirmed on the bases of clinical and laboratory investigations of urine samples and a positive bacterial culture from urine of suspected camels (*n* = 74). Camels that have a signs of UTI without positive bacterial culture were excluded from the study (2 females).

### Physical examination

In order to make a clinical diagnosis, all camels underwent a thorough physical examination (Rosenberger, 1979), which included general behavior and condition, auscultation of the heart, lungs, rumen and intestine, measurement of heart rate, respiratory rate and rectal temperature, swinging auscultation and percussion auscultation of both sides of the abdomen, and rectal examination.

### Hematological and Biochemical analysis

Blood samples were collected from the jugular vein in plain tubes from all camels upon arrival to the hospital. Plasma and serum were obtained from blood samples and processed according to Coles (1986) protocol.

### Serum analysis

The serum samples were tested using an automated biochemical analyzer (VetScan VS2; Abaxis, Union City, California, USA) to determine the concentration of total protein, albumin, globulin, blood urea nitrogen (BUN) and creatinine.

### Preparation of erythrocyte hemolysate

Immediately after collection, blood samples were centrifuged at 1,500 rpm for 15 min at 4 °C. The plasma and buffy coats were removed by aspiration. The sediment containing blood cells was washed three times by resuspending in isotonic phosphate-buffered saline (PBS, containing 8.9 mM Na_2_HPO_4_, 1.1 mM Na_2_HPO_4_, and 140 mM Nacl, pH 7.4) followed by re-centrifugation (1,500 rpm for 10 min at 4 °C) and removal of the supernatant fluid and the buffy coats. The crude red cells were lysed in nine volumes of ice-cold distilled water to prepare a 10% erythrocyte hemolysate.

### Erythrocytic glutathione (GSH)

GSH concentration in the RBC hemolysate was measured using the method of Beutler, Duron & Kelly (1963); this method is based on the development of a stable yellow color when 2-nitrobenzoic acid is added to sulfhydryl compounds. The amount of reduced product, thionitrobenzene, was measured at 412 nm and expressed as mmol/g Hb. (Shimadzu AA-6800 atomic absorption spectrophotometer; Shimadzu, Kyoto, Japan).

### Superoxide Dismutase (SOD)

SOD activity was estimated in the RBC hemolysate according to the method described by Misra & Fridovich (1972). This method is based on the ability of SOD to inhibit the autoxidation of epinephrine to adrenochrome in an alkaline medium (pH 10.2). The optical density (OD) was measured at 480 nm and expressed as U/mg Hb (Shimadzu AA-6800 atomic absorption spectrophotometer; Shimadzu, Kyoto, Japan).

### Catalase (CAT)

CAT activity was measured in the RBC hemolysate by the method of Beers & Sizer (1952). Decomposition of H_2_O_2_ was followed directly by the decrease in absorbance at 240 nm, and the difference in absorbance per min/mg Hb was taken as a measure of the CAT activity (Shimadzu AA-6800 atomic absorption spectrophotometer; Shimadzu, Kyoto, Japan).

### Malondialdehyde (MDA)

Lipid peroxidation in RBC hemolysate and serum was estimated as thiobarbituric acid reactive substances (TBARS) according to Placer, Cushman & Johnson (1966). The method is based on forming a color complex between the products of lipid peroxidation and thiobarbituric acid (TBA). Briefly, 0.2 mL of serum or RBC hemolysate was added to 1.3 mL of 0.2 mol/l Tris, 0.16 mol/l KCl buffer (pH 7.4). TBA (1.5 mL) was added and the mixture was heated in a boiling water bath for 10 min. After cooling, 3 mL of pyridine–butanol (3:1 v/v) and 1 mL of 1 mol/l NaOH were added. The absorbance was read at 548 nm against bi-distilled water as a blank. In this assay, 1,1,3,3-tetramethoxypropane was used as a standard. Lipid peroxidation in the RBC hemolysate was expressed as nmol of erythrocytic malondialdehyde (eMDA)/g Hb. Lipid peroxidation in serum was expressed as *nmol* of serum malondialdehyde (sMDA)/g serum protein (Shimadzu AA-6800 atomic absorption spectrophotometer; Shimadzu, Kyoto, Japan).

### Acute phase proteins and IL-6

Hp and SAA were measured with a commercially available ELISA kit (Tridelta Development Plc, Wicklow, Ireland), according to the manufacturer’s instructions (non-species specific kits). The analytical sensitivities of these tests in plasma have been determined as 0.3 µg/mL for SAA and 0.0156 mg/mL for Hp by the manufacturer (Tridelta Development Plc, Wicklow, Ireland). Fibrinogen was measured by heat precipitation-refractometry method as described by Duncan, Prasse & Mahaffey (1994).

IL-6 level was determined from undiluted serum samples using commercially available ELISA Kits (Biosource, Diagnostic Corporation, Nivelles, Belgium). The plates read at 450 nm on a computerized automated microplate ELISA reader (ELX800G; BioTek Instruments, Winooski, Vermont, USA). Cp activity was measured according to its phenylenediamine oxidase activity (Shimadzu AA-6800 atomic absorption spectrophotometer; Shimadzu, Kyoto, Japan) according to the method described by Sunderman & Nomoto (1970).

### Urinalysis

Urine samples were obtained via catheterization in females and via free flow into sterile plastic specimen cups in males and consequently evaluated for color, transparency and odor. The urine was also assessed using a strip test (Combur^9^-Test; Roche, Basel, Switzerland). Smears of the urine sediment were stained with Gram stain and examined microscopically, and a urine sample was cultured bacteriologically on blood agar, nutrient agar and MacConkey agar for 48 h at 37 °C. The isolated bacteria were identified using a VITEK2 Compact (bioMerieux, Marcy-l’Étoile, France). Antibacterial susceptibility tests were performed using the standard methods of the US National Committee for Clinical Laboratory Standards (1997).

### Statistical analysis

All statistical analysis were performed with commercial statistical software (SAS v.9.3, Cary, NC and MedCalc V.13, Mariakerke, Belgium). Because of the small size of the control group and non-normally distributed markers in camels with UTI, each blood biomarker was assessed using non-parametric analysis (Wilcoxon Mann–Whitney) to compare the data between cases and controls, and between camels with treatment success or failure.

For each marker of potential interest for the diagnosis of treatment failure (*P* value less than 0.05), a crude univariate analysis of the biomarker as a predictor of treatment failure was performed so as to avoid any false assumption concerning the marker distribution determining the Area under the receiver operating characteristic (ROC) curve and the Youden’s index J which minimizes misclassification (J = Max (Se + Sp−1)). Internal validation of the J value for each marker was performed using internal resampling with replacement (bootstrap sample of 1,000 datasets) in order to determine the interval, which contained 95% of the observed J values.

The cutoff for each marker was subsequently chosen in observed interval (using a round value included in the 95% CI). This cutoff was used to create dichotomous covariates with the referent being the normal category (ie not associated with a negative outcome) in order to assess these variables in logistic models. A correlogram was then obtained using a Spearman rank correlation (*r_s_*) to avoid multicolinearity in model building. The correlation was considered significant for *r_s_* ⩾ 0.5. The correlated biomarkers were not put in the same model to avoid model instability. Two logistic regression models were then built using oxidative stress markers (sMDA) and inflammatory markers (SAA or haptoglobin). The sex was also included as a covariate. A first model was built with sex, sMDA and haptoglobin as potential covariates. The second model was built with sex, sMDA and SAA as potential covariates. Each model was built backward (SAS Logistic procedure) and the fit of the model was assessed using the Hosmer and Lemeshow test (Hosmer et al., 1997). The area under the ROC curve of each model was compared using a non-parametric Mann–Whitney U-test (DeLong, DeLong & Clarke-Pearson, 1988).

### Treatment protocol

The camels with UTI received one of the following antibiotic therapies for 10–21 days according to the results of sensitivity tests, amoxicillin (Clamoxyl, GlaxoSmithKline, Brentford, UK) IM once daily, 7 mg/kg (*n* = 38), procaine penicillin G (Pfizer Animal Health, Lee’s Summit, Missouri, USA), 7 mg/kg every 8 h (*n* = 21) and Ceftiofur (Excenel RTU; Pfizer, South San Francisco, California, USA) 2.2 mg/kg, IM (*n* = 15). Moreover, all diseased camels received flunixin meglumine (Finadyne; Schering-Plough Corporation, Kenilworth, New Jersey, USA) 1.1 mg/kg body weight IV for three days. All diseased camels were treated with 10 L of dextrose-saline (dextrose 5% and saline 0.9%) administered IV in a slow drip, daily for 2 to 3 days.

The camels with UTI were further categorized into two groups according to the response to treatment (the treatment based on urine culture and sensitivity tests for isolated bacteria and selection of the proper antibiotics), the success group (*n* = 33) and the other failure one (*n* = 41). The success to treatment was based on the absence of clinical signs, clinical examination of camels and negative urine culture. The response to treatment varies from 5–14 days in camels with UTI.

## Results

### Clinical picture of UTI in camel

Thirty-three males and forty-one females had a UTI diagnosis. The main clinical signs that were observed were anorexia (*n* = 64), dysuria (*n* = 74), stranguria, pollakiuria (*n* = 74), blood-tinged urine (*n* = 65) and abdominal pain (*n* = 70). The rectal examination of affected animals showed severe pain sensation during bladder palpation and resistance to the examination. No clinical abnormalities were detected in other parts of the urinary tract.

### Urine analysis findings

Urine analysis for UTI cases revealed the presence of protein. The microscopical examination of urine revealed hematuria and pyuria. The isolated bacteria were *E. coli* (*n* = 34), *Corynebacterium renale* (*n* = 31) and mixed bacterial culture with different types of bacteria including *Corynebacterium* with other bacteria as *Staphylococci, Streptococci* and *Proteus* (*n* = 9).

### Hematological and biochemical findings

The concentrations of eMDA, sMDA were significantly (*P* < 0.0001) higher in diseased camels when compared to healthy ones. Moreover, catalase, super oxide dismutase and glutathione levels were significantly (*P* < 0.0001) lower in diseased camels when matched with the same levels in control group (Table 1).

**Table 1:** Descriptive results and univariate analysis of blood biomarkers in camels with clinical diagnosis of UTI and in healthy camels.

	Clinical cases (*n* = 74)				Healthy cases (*n* = 15)				
Variable	Mean	Median	Minimum	Maximum	Mean	Median	Minimum	Maximum	P^*^ value
Age (Year)	7.11	7.00	5.40	10.20	6.03	5.90	3.80	7.80	0.009
eMDA (nmol/g Hb)	180.80	180.36	104.23	222.36	109.41	109.36	101.36	113.54	<0.0001
sMDA (nmol/g protein))	19.01	19.30	10.10	24.64	10.85	11.11	10.23	11.60	<0.0001
CAT *(U/mg Hb*)	11.36	10.33	8.56	20.10	15.67	15.60	15.20	16.30	<0.0001
GSH (mmol/g Hb)	4.10	3.80	2.20	9.11	6.78	6.80	6.30	7.20	<0.0001
SOD (U/mg Hb)	3.73	3.45	2.12	8.12	4.98	4.90	4.50	5.80	<0.0001
Cp (g/L)	1.06	1.09	0.08	3.60	0.09	0.09	0.08	0.10	<0.0001
HP (g/L)	2.45	2.34	0.10	6.50	0.31	0.31	0.26	0.35	0.0002
SAA (µg/mL)	15.70	13.77	8.85	28.60	9.50	9.60	8.90	9.90	<0.0001
Fibrinogen (g/L)	4.28	4.20	2.40	7.60	3.27	3.30	2.80	3.60	<0.0001
Total protein (g/L)	6.85	6.32	5.38	8.90	6.05	5.90	5.40	7.20	0.12
Albumen (g/L)	2.64	2.57	2.30	3.30	3.13	3.10	2.80	3.60	<0.0001
Globulin (g/L)	4.21	3.45	2.50	6.42	2.91	2.90	2.40	3.80	<0.0001
BUN (mg/dl)	11.52	11.23	9.69	16.32	11.16	11.30	9.60	12.80	0.64
Créatinine (mg/dL)	0.88	0.87	0.50	1.23	0.95	0.94	0.80	1.20	0.11
IL-6 (pg/mL))	15.08	14.43	11.23	21.36	12.35	12.36	10.23	13.87	0.003

**Notes.**

UTI Urinary Tract Infection eMDA erythrocytic malondialdehyde sMDA Serum malondialdehyde SOD super oxide dismutase GSH glutathione CAT catalase Hp Haptoglobin SAA Serum Amyloid A Cp Ceruloplasmin BUN blood urea nitrogen Fb Fibrinogen IL-6 interleukin 6

* *P*-value resulting from non-parametric Wilcoxon Mann-Whitney test.

Furthermore, Hp, SAA, Fb, Cp, albumin, globulin and IL-6 level were significantly higher in diseased camels when compared with their values in control group (Table 1).

Regarding the success or failure to treatment, there were 41 failure cases versus 33 success camels to the selected antibiotics (Table 2). The success or failure to treatment therapy was significantly correlated with the levels of eMDA sMDA (*P* < 0.0001), as well as the levels of catalase, super oxide dismutase, and glutathione (*P* < 0.0001). In addition, the success or failure to treatment was significantly correlated with the levels of Hp (*P* < 0.005), SAA (*P* < 0.0001), Fb (*P* < 0.002), total protein (*P* < 0.0001), globulin (*P* < 0.0001) and IL-6 level (*P* < 0.0004) as shown in Table 2.

**Table 2:** Description of the variables depending on the success or failure with the treatment.

	Success cases (*N* = 33)				Failure cases (*N* = 41)				
Variable	Mean	Median	Minimum	Maximum	Mean	Median	Minimum	Maximum	P^*^-value
Age (Year)	6.72	6.80	5.50	8.40	7.43	7.20	5.40	10.20	0.01
eMDA (nmol/g Hb)	163.99	171.36	104.23	182.36	194.66	211.36	105.36	222.36	<0.0001
sMDA (nmol/g protein))	16.65	17.36	10.11	19.36	20.92	22.58	10.10	24.64	<0.0001
CAT (U/mg Hb)	12.15	11.60	9.30	20.10	10.72	9.40	8.56	16.36	
GSH (mmol/g Hb)	4.83	4.20	3.60	9.11	3.51	3.10	2.20	7.32	<0.0001
SOD (U/mg Hb)	4.25	3.80	3.10	8.12	3.32	3.10	2.12	7.25	<0.0001
Cp (g/L)	1.23	0.93	0.08	3.60	0.93	1.09	0.08	3.50	0.15
HP (g/L)	3.30	3.60	0.10	6.50	1.78	1.30	0.23	5.20	0.005
SAA (µg/mL)	19.67	19.70	8.85	28.60	12.51	11.86	8.96	25.30	<0.0001
Fibrinogen (g/L)	4.72	4.60	2.94	7.60	3.92	3.90	2.40	5.80	0.002
Total protein (g/L)	5.70	5.70	5.38	6.34	7.77	7.90	5.42	8.90	<0.0001
Albumen (g/L)	2.62	2.56	2.30	3.20	2.65	2.58	2.38	3.30	0.3
Globulin (g/L)	3.08	3.12	2.50	3.72	5.12	5.24	2.97	6.42	<0.0001
BUN (mg/dL)	10.89	10.80	9.78	12.40	12.02	11.54	9.69	16.32	0.003
Creatinine (mg/dL)	0.84	0.82	0.50	1.20	0.91	0.90	0.59	1.23	0.07
IL-6 (pg/mL))	16.69	18.25	11.23	21.36	13.79	13.69	11.23	19.36	0.0004

**Notes.**

eMDA erythrocytic malondialdehyde sMDA Serum malondialdehyde SOD super oxide dismutase GSH glutathione CAT catalase Hp Haptoglobin SAA Serum Amyloid A Cp Ceruloplasmin BUN blood urea nitrogen Fb Fibrinogen IL-6 interleukin 6

* *P*-value resulting from non-parametric Wilcoxon Mann-Whitney test.

Globulin (AUC = 0.94), Total protein (AUC = 0.0.938), MDA (0.867) were the most accurate in predicting treatment outcome in camels with UTI. While catalase (AUC = 0.72), fibrinogen (AUC = 0.717), Blood urea nitrogen (AUC = 0.712) and IL-6 (AUC = 0.751) were moderate in predicting treatment outcome in diseased camels as presented in Table 3.

**Table 3:** The optimized cutoff of all selected biomarkers and their respective sensitivity (Se) and specificity (Sp) to detect failure or success.

Variables	Criterion observed	Se	Sp	Bootstrap CI^a^	Criterion used^b^	AUC^c^	95% CI^d^	P^*^ value (failure vs success)
eMDA (nmol/g Hb)	>182.36	0.850	1.0	180.34–182.36	180	0.867	0.767–0.935	<0.0001
sMDA (nmol/g protein))	>19.36	0.854	1.0	18.4–19.36	19	0.866	0.767–0.934	<0.0001
CAT (U/mg Hb)	≤9.4	0.512	0.909	9.1–10.69	9.4	0.72	0.603–0.818	0.002
GSH (mmol/g Hb)	≤3.6	0.781	0.909	3.3–3.6	3.5	0.854	0.753–0.925	<0.0001
SOD (U/mg Hb)	⩽3.4	0.805	0.879	3.1–3.4	3	0.817	0.711–0.898	<0.0001
Cp (g/L)	≤0.13	0.390	0.879	0.08–0.13	NU	0.600	0.479–0.712	0.15
HP (g/L)	≤3.39	0.902	0.606	1.34–3.39	3	0.698	0.581–0.800	0.005
SAA (µg/mL)	≤15.6	0.927	0.879	13.8–15.6	15	0.853	0.751–0.924	<0.0001
Fibrinogen (g/L)	≤4.23	0.756	0.667	3.4–4.5	4	0.717	0.600–0.815	0.002
Total protein (g/L)	>6.34	0.878	1.0	5.8–6.34	6.0	0.938	0.857–0.981	<0.0001
Albumen (g/L)	>2.53	0.732	0.455	2.36–2.79	NU	0.571	0.451–0.686	0.3
Globulin (g/L)	>3.72	0.854	1.0	3.27–3.72	3.5	0.940	0.859–0.982	<0.0001
BUN (mg/dL)	>12.3	0.488	0.939	10.6–12.3	12	0.712	0.595–0.811	0.003
Creatinine (mg/dL)	>0.95	0.488	0.758	0.68–1.06	0.95	0.626	0.506–0.736	0.07
IL–6 (pg/mL)	≤15.47	0.902	0.727	12.36–15.47	15	0.751	0.637–0.844	0.0004

**Notes.**

eMDA erythrocytic malondialdehyde sMDA Serum malondialdehyde SOD super oxide dismutase GSH glutathione CAT catalase Hp Haptoglobin SAA Serum Amyloid A Cp Ceruloplasmin BUN blood urea nitrogen Fb Fibrinogen IL-6 interleukin 6 NU not used since univariate analysis was higher than 0.1 or because not interesting per se. Se sensitivity of the threshold indicates the number of cases positive (with treatment failure) for the tests/total number of cases Sp specificity of the threshold indicates the number of cases with treatment success with a negative test/total number of treatment success

* *P*-value resulting from non-parametric Wilcoxon Mann-Whitney test.

a A internal resampling with replacement using bootstrap technique was used to derive the interval which contained 95% of the observed J based on these 1,000 datasets.

b The cutoff chosen for further modeling was chosen within interval of distribution of J as a rounded value to be used in a dichotomous covariate in multivariate analysis.

c AUC: area under the receiver operating characteristic curve. The AUC was derived from the non-parametric ROC curve obtained using the initial dataset.

d 95% confidence interval for the AUC.

### Predictive models

**Figure 1:**
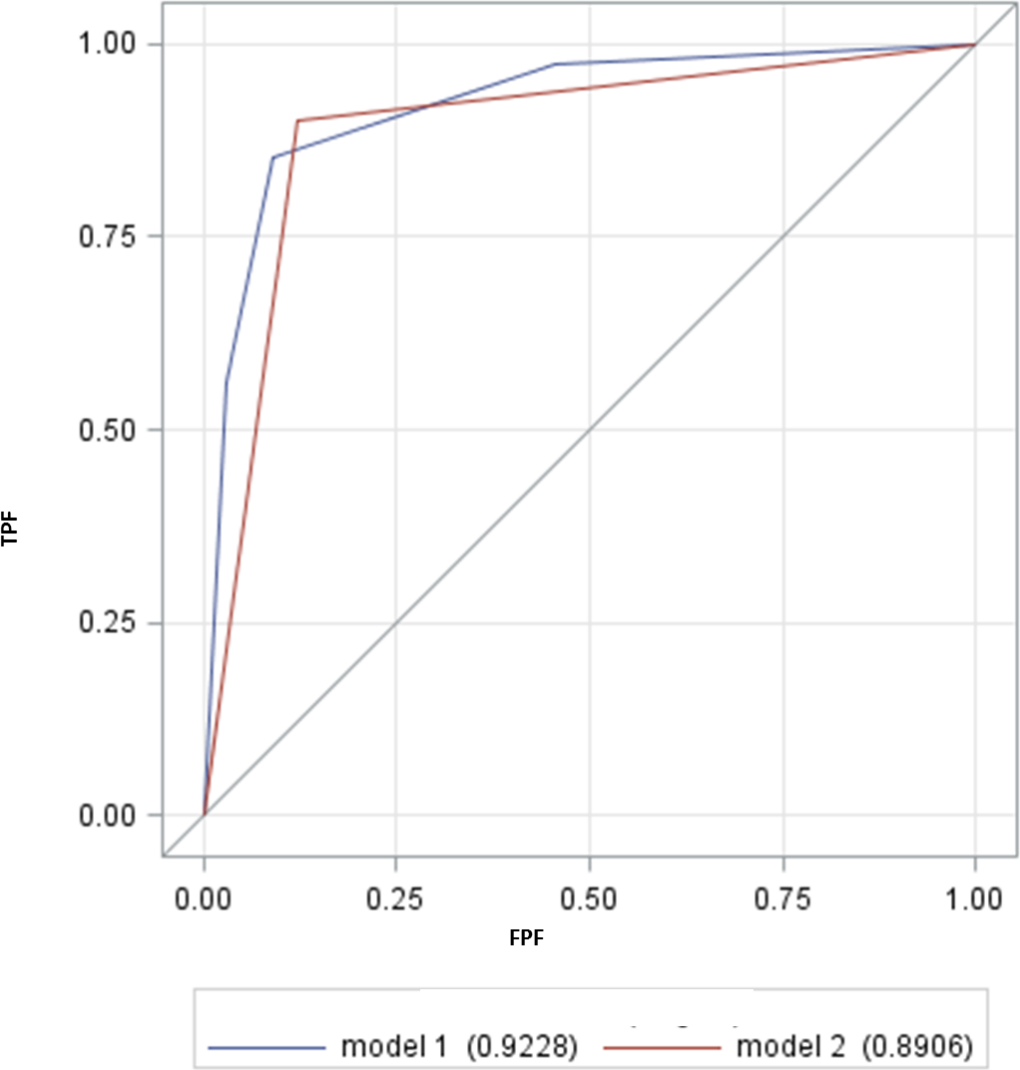
The receiver operating characteristic curves (ROC) of 2 different models. TPF: true positive fraction; FPF: false positive fraction. Model 1 was the model including sex and sMDA as covariates. Model 2 included SAA as predictor of treatment failure.There was no difference between the 2 AUC (Mann-Whitney *U* test, *P* = 0.54).

Two predictive models were built (Table 4). Both models had a good predictive ability in differentiating the success from failure camels to treatment program (Model 1, AUC = 0.92; 95% CI [0.86–0.98] and Model 2 AUC = 0.89; 95% CI [0.82–0.96]). The sensitivity (Se: proportion of cases with failure) were 85.4% and 90.2% and the specificity (Sp: proportion of successfully treated camel correctly classified) were 90.9% and 87.9% for model 1 and 2 respectively. There were no significant differences between the predictive ability of both models (*P* = 0.54; Fig. 1).

**Table 4:** The two logistic regression models predicting the probability of treatment failure in camels with cystitis.

Model	Variable	Coefficient	SE	OR	95% CI	Joint *P*-value
Model 1	Intercept	−0.940	0.0.513	–	–	0.067
	Female	Ref	Ref	Ref	Ref	0.04
	Male	−1.734	0.847	0.17	0.034–0.928	
	Low sMDA^a^	Ref	Ref	Ref	Ref	<0.0001
	High sMDA^a^	4.341	0.868	76.76	14.00–420.98	
Model 2	Intercept	−1.981	0.533	–	–	0.0002
	Low SAA^b^	Ref	Ref	Ref	Ref	<0.0001
	High SAA^b^	4.206	0.749	67.06	15.44–291.28	

**Notes.**

OR Odds Ratio

a The cutoff chosen was 19 nmol/g protein (as shown in Table 3).

b The cutoff used was 15 µg/mL as shown in Table 3.

## Discussion

The diagnostic and prognostic importance of acute phase proteins, and oxidative stress biomarkers in cases of UTI in dromedary camels were studied. The presented clinical signs of diseased camels are in concurrence with the clinical picture of cows suffered UTI (Van Metre & Divers, 2002; Yeruham et al., 2006; Radostits et al., 2007). Despite the limited number of cases, we were able to find potentially interesting diagnostic and prognostic markers that need to be confirmed in future clinical studies.

Oxidative stress is any disturbance in the normal redox state of cells that will cause toxic effect due to production of peroxides and free radicals leading to damage of all components of the cell, including proteins, lipids, and DNA (Kowaltowski & Vercesi, 1999). Thus, oxidative stress can cause disruptions in normal mechanisms of cellular ability to detoxify the reactive intermediates or to repair the resulting damage (Lands et al., 2000).

A complex association exists between oxidative stress and inflammation as documented previously and confirmed in this investigation. Oxidative stress is a consequence of the imbalance between reactive oxygen species (ROS) and production and antioxidant capacity. This can occur because of either heightened ROS generation, impaired antioxidant system, or a combination of both. In the presence of oxidative stress, uncontained ROS attack, modify, and denature functional and structural molecules leading to tissue injury and dysfunction (Vaziri, 2008).

Data described in this study provide a reliable biochemical evidence for the generation of circulating oxidative stress as detected by enhanced lipid peroxidation (sMDA and eMDA) and decreased serum levels of the enzymatic (SOD, CAT) and non-enzymatic (GSH) antioxidant markers in dromedary camels suffering UTI. It was reported that MDA levels were increased in a varieties of inflammatory conditions like acute and chronic cystitis in camels (Abd Ellah, Khamis & Elnisr, 2012), UTI in human patients (Kurutas et al., 2005), liver abscess in camels (El-Deeb & Fouda, 2013), mastitis in does (El-Deeb, 2013) pneumonia in calves (El-Bahr & EL-Deeb, 2013). In addition, levels of lipid hydroperoxide were increased in erythrocytes isolated from dairy cows with acute mastitis (Castillo et al., 2006). However, parturition and early lactation may in itself be associated with increased lipid peroxidation, as measured by TBARS/MDA (Bernabucci et al., 2002; Bernabucci et al., 2005; Castillo et al., 2005; Castillo et al., 2006). In bronchopneumonic calves, it was found that isolated granulocytes produced ten times as much ${\mathrm{O}}_{2}^{-.}$ and have lower plasma superoxide dismutase compared with healthy calves (Ledwozyw & Stolarczyk, 1992). Another study found that isolated neutrophils released from diseased animals produce large amounts of NO^−^ and myeloperoxidase, which in combination may result in nitrotyrosine formation (i.e., protein damage) (Wessely-Szponder et al., 2004). Marked decreases in ascorbate concentrations have been found in dairy cattle with subclinical forms of mastitis caused by *Staphylococcus aureus, Streptococcus agalactiae* or *E. coli* in comparison with healthy controls (Kleczkowski et al., 2005). This has also been demonstrated by Ranjan et al. (2005), both in acute and subclinical mastitis.

Interestingly, it was discovered that the levels of eMDA & sMDA (Sensitivity 85%, Specificity 100% AUC = 0.86) were considered as sensitive and specific biomarkers differentiating diseased from non-diseased camels and also the success from failure cases as shown in Tables 1 and 2. These results are in agreement with those reported in patients with acute appendicitis (Kavakli, Erel & Becel, 2011), male infertility (Amarasekara et al., 2014), diagnosis and prognosis of atherosclerosis (Heinecke, 2003) and in diagnosis of patients with complicated and uncomplicated parapneumonic pleural effusions (Tsilioni et al., 2011).

In this study, there was a significant increase in Hp, SAA, Cp, and Fb levels in camels with UTI when compared to healthy ones. The primary trail leading to significant elevation in APPs in diseased camels may involve initial release of pro-inflammatory cytokines by macrophages at the site of inflammation of urinary tract (Glass et al., 2003). Their circulating levels may also be related to the severity of the response to infection, and thus may provide valuable quantifiable biochemical indicators of the inflammatory response. Inflammation of urinary tract as detected in this study induce strong acute phase responses manifested by elevated levels of SAA, HP, Fb and Cp. The specific type of APPs and the time course for alterations in these proteins vary in different species on the basis of the initiating disorder or underlying inflammatory process (Feldman, Zinkl & Jain, 2000).

The values for serum Hp of apparently healthy dromedary camels in this study was 0.26–0.35 g/L, which is in agreement to values reported for camel before and after transportation (Baghshani et al., 2010). It is higher than the reference value which was reported for healthy cows (0.022–0.047 g/L) by Salonen et al. (1996) whereas it is lower than reported values for healthy horses (1.43 ± 0.68 g/L by Kent & Goodall (1991)). The concentration of serum Hp in this study was higher than the value reported for sheep (Nowroozi-Asl, Nazifi & Bahari, 2008; Razavi et al., 2010; Razavi et al., 2011; Mohebbi et al., 2010). In this study, the values for SAA of apparently healthy dromedary camels was 8.9–9.9 µg/mL which is in agreement to the values reported for camel before and after transportation (Baghshani et al., 2010). However, it was higher in comparison with the values which was reported for cattle (Ansari-Lari et al., 2008; Nazifi, Khoshvaghti & Gheisari, 2008; Nazifi et al., 2008b; Nazifi et al., 2009a; Nazifi et al., 2009b; Nazifi et al., 2010a; Nazifi et al., 2010b) and sheep (Mohebbi et al., 2010; Razavi et al., 2011).

It was previously reported that APP concentrations are elevated in many diseases with different pathogeneses (Murata, Shimada & Yoshioka, 2004). The fact causes APPs to have poor specificity in detecting the cause for a particular disease but some studies have been performed to increase the specificity of APPs, using group analysis of APPs (Gruys et al., 2005). The elevated levels of acute phase proteins was previously detected in several bacterial infections including, UTI in mice (Erman et al., 2012) *Escherichia coli* mastitis (Suojala et al., 2008), Subclinial Staphylococcus aureus mastitis (Eckersall et al., 2006), *Mannheimia haemolytica* (Gånheim et al., 2003), *Pasteurella multocida* (El-Deeb & Tharwat, 2015). Similarly, it was detected to be elevated in various inflammatory conditions including chronic respiratory disease (Huzzey et al., 2009; Chan et al., 2010), metritis (Tabrizi et al., 2008), lameness (Kujala, Orro & Soveri, 2010; Smith, Kauffold & Sherman, 2010), and traumatic reticuloperitonitis (Nazifi et al., 2009a).

In this study, there was significant increase in the levels of IL-6, in UTI group when compared to healthy ones. The elevated levels of IL-6 might be attributed to the inflammation of the urinary tract. Such inflammatory condition induce the release of cytokines under the effect of which, APPs are synthesized in liver (Radostits et al., 2007).

Interestingly, it was detected from the first model including sex, sMDA and Hp that this model showed a good predictive ability in differentiating the success from failure cases (AUC = 0.92). In the same concern, it was observed from the second model involved sex, SAA and sMDA that this model declared a good predictive ability for differentiating the success from failure cases (AUC = 0.89). Moreover it was also detected that IL-6 could also be used as a biomarkers for UTI in camels and also differentiating the success from failure cases (Sensitivity 90%/Specificity 72%). Also, globulin could be used as an additional biomarker for UTI in camels and also differentiating the success from failure cases (Sensitivity 85%/Specificity 100%).

APPs could be used as a prognostic tool, with the magnitude and duration of the APR reflecting the severity of infection (Petersen, Nielsen & Heegaard, 2004). From the present study, it could be concluded that oxidative stress biomarkers (MDA) and acute phase proteins (SAA) could be used as a diagnostic and prognostic biomarkers in dromedary camels with UTI.

## Supplemental Information

10.7717/peerj.1363/supp-1 Supplemental Information 1 Dataset used for the study
